# Effects of Multiplicative Noise in Bistable Dynamical Systems

**DOI:** 10.3390/e27020155

**Published:** 2025-02-02

**Authors:** Sara C. Quintanilha Valente, Rodrigo da Costa Lima Bruni, Zochil González Arenas, Daniel G. Barci

**Affiliations:** 1 PPG-CompMat, Universidade do Estado do Rio de Janeiro, Rua São Francisco Xavier 524, Rio de Janeiro 20550-013, RJ, Brazil; saravalenteq@gmail.com (S.C.Q.V.); bruni.r.c.l@gmail.com (R.d.C.L.B.); 2 Departamento de Matemática Aplicada, Universidade do Estado do Rio de Janeiro, Rua São Francisco Xavier 524, Rio de Janeiro 20550-013, RJ, Brazil; 3 Departamento de Física Teórica, Universidade do Estado do Rio de Janeiro, Rua São Francisco Xavier 524, Rio de Janeiro 20550-013, RJ, Brazil

**Keywords:** stochastic dynamic, Langevin equations, multiplicative noise, decay rates, bistable systems

## Abstract

This study explores the escape dynamics of bistable systems influenced by multiplicative noise, extending the classical Kramers rate formula to scenarios involving state-dependent diffusion in asymmetric potentials. Using a generalized stochastic calculus framework, we derive an analytical expression for the escape rate and corroborate it with numerical simulations. The results highlight the critical role of the equilibrium potential $U_{\mathrm{eq}}\left(x\right)$, which incorporates noise intensity, stochastic prescription, and diffusion properties. We show how asymmetries and stochastic calculus prescriptions influence transition rates and equilibrium configurations. Using path integral techniques and weak noise approximations, we analyze the interplay between noise and potential asymmetry, uncovering phenomena such as barrier suppression and metastable state decay. The agreement between numerical and analytical results underscores the robustness of the proposed framework. This work provides a comprehensive foundation for studying noise-induced transitions in stochastic systems, offering insights into a broad range of applications in physics, chemistry, and biology.

## 1. Introduction

Dynamical systems often exhibit multiple local equilibrium configurations separated by potential barriers. The primary mechanism for the decay of these states is thermal activation over a potential barrier. This phenomenon has been widely investigated due to its significance in diverse fields such as chemistry, physics, and biology [1].

A simple representation for thermal activation considers a classical particle in a bistable potential, U(x), undergoing stochastic dynamics governed by a Langevin equation with additive white noise. A key quantity in this framework is the escape rate, which characterizes the particle’s transition from a local minimum of the potential.

Kramers’s pioneering work [2] provides an elegant formula for the escape rate in symmetric potentials:

(1) $$r_{\mathrm{add}}=\frac{\sqrt{\omega_{\min}\left|\omega_{\max}\right|}}{2\pi }e^{-\frac{\Delta U}{\sigma^{2}}},$$

where $\Delta U=U\left(x_{\max}\right)-U\left(x_{\min}\right)$ is the barrier height, with $x_{\max}$ and $x_{\min}$ denoting the positions of the maximum and the minimum of the potential, respectively. The noise intensity, represented as $\sigma^{2}∼k_{B}T$, where $k_{B}T$ is the thermal energy, quantifies the strength of the stochastic fluctuations in the system, and $\omega_{\min}=U^{\prime \prime }\left(x_{\min}\right)/m$ and $\omega_{\max}=U^{\prime \prime }\left(x_{\max}\right)/m$ are frequencies associated with a parabolic approximation of the potential energy. $U^{\prime \prime }\left(x_{\min}\right)$ and $U^{\prime \prime }\left(x_{\max}\right)$ are the local curvatures of the potential at the minimum and the maximum and *m* is the mass of the particle. This result holds in the weak noise regime ($\sigma^{2}\ll \Delta U$).

Recently, we extended Kramers’s formula, given by Equation (1), to systems with multiplicative noise [3]. In such systems, the stochastic dynamics are driven by a state-dependent diffusion function, g(x). This state-dependent nature add further challenges and require a careful treatment using stochastic calculus, where the choice of interpretation, such as those by Itô, Stratonovich, or others, can influence the resulting dynamics [4,5,6]. Understanding Kramers’s rate in the context of multiplicative noise is not merely of theoretical interest but has practical implications. A significant application is stochastic resonance, a phenomenon in which noise amplifies the response of a nonlinear system to weak periodic signals [7].

In addition, evidence of heterogeneous diffusion has been observed in very different systems such as gene expression processes [8], microparticles in confined geometries [9,10], and colloids [11,12]. From a theoretical perspective, stochastic systems with multiplicative noise, i.e., with heterogeneous diffusion, have been investigated using a variety of approaches. These range from numerical simulations of Langevin equations [13,14,15,16] to mean-field approximations of Fokker–Planck equations [17] and further to path integral techniques [18,19,20,21], which are particularly well suited for exploring symmetries, conserved quantities, and fluctuation theorems [22].

While prior studies of Kramers’s escape rate and its generalizations have primarily addressed symmetric potentials, real-world systems often exhibit asymmetric potential landscapes. Asymmetries introduce new complexities, such as unequal barrier heights and different curvatures at the minima, which significantly impact escape dynamics. This requires further generalization of classic escape rate formulas.

In this work, we derive an explicit analytic formula for the escape rate in stochastic systems with multiplicative noise under asymmetric potentials. We use a broad stochastic framework to define the Langevin equation, which includes the most common interpretations of stochastic integration. Our analysis highlights how asymmetry modifies the escape rate and its dependence on the stochastic prescription. The main result of this paper is the explicit analytic expression for the decay rate

(2) $$r=\frac{g{\left(x_{m}\right)}^{2}}{2}\left(\frac{\sqrt{{\tilde{\omega }}_{a}\left|{\tilde{\omega }}_{m}\right|}}{2\pi }e^{-\frac{\Delta U_{\mathrm{eq}}^{a}}{\sigma^{2}}}+\frac{\sqrt{{\tilde{\omega }}_{b}\left|{\tilde{\omega }}_{m}\right|}}{2\pi }e^{-\frac{\Delta U_{\mathrm{eq}}^{b}}{\sigma^{2}}}\right)$$

where $\Delta U_{\mathrm{eq}}$ is the height of the equilibrium potential barrier related to each of the minima x=a or x=b, $\tilde{\omega }$s are the local curvatures of the equilibrium potential at the minima or the maximum of the potential, and $g(x_{m})$ is the diffusion function evaluated at the local maximum of $U_{\mathrm{eq}}$. In this case, the square of the diffusion function is playing the role of the inverse of the mass, since the actual frequency is proportional to $g^{2}\tilde{\omega }$. The main feature of this expression is that it depends neither on the details of the equilibrium potential nor on those of the diffusion function. Instead, it relies solely on the local properties of these quantities, such as the height of the barrier and the local curvatures at the minima and maximum. On the other hand, all the information about the specific stochastic prescription defining the multiplicative noise process is encapsulated in the equilibrium potential. This potential incorporates not only the original bare potential but also information on the multiplicative noise fluctuations. Moreover, we observe that both minima, the global and the metastable one, contribute to the decay rate, independently of the initial conditions of the process.

We calculate the escape rate using the conditional probabilities of state transitions over time. The computation employs path integral techniques, utilizing an instanton–anti-instanton expansion valid in the weak noise limit and long times. Our approach closely follows the methods in [3,23]. Additionally, we perform extensive numerical simulations of the Langevin equation for specific potential and diffusion functions. These results enable us to evaluate the validity and limitations of our approximations.

The structure of this paper is as follows. Section 2 present the theoretical framework for analyzing asymmetric potentials in the presence of additive and multiplicative noise. Section 3 reviews the path integral approach to stochastic processes, emphasizing its application to escape rate calculations. In Section 4, we derive the weak noise approximations for escape rates in asymmetric potentials, presenting the main result of this paper given by Equation (2). Numerical simulations and comparisons with analytic results are presented in Section 5. Finally, we provide a summary of our findings and discuss potential applications in Section 6.

## 2. A Simple Representation of State-Dependent Diffusion

Consider a one-dimensional conservative system characterized by a potential energy function, U(x), that has a double-well structure. The system interacts with a thermal bath described by a state-dependent diffusion function, g(x). The overdamped Langevin equation is given by

(3) $$\frac{dx(t)}{dt}\overset{\alpha }{=}f\left(x\left(t\right)\right)+g\left(x\left(t\right)\right)\eta \left(t\right),$$

where η(t) is a Gaussian white noise, thus satisfying

(4) $$\langle \eta (t)\rangle =0\;\;,\;\;\;\langle \eta \left(t\right)\eta \left(t^{\prime }\right)\rangle =\sigma^{2}\delta \left(t-t^{\prime }\right)\;.$$

The drift force and the diffusion function are represented by f(x) and g(x), respectively, which are arbitrary smooth functions of *x*, and σ measures the noise intensity. The symbol α over the equal sign indicates that the Langevin equation is interpreted in the generalized Stratonovich [24] convention or α-prescription [25]. In this prescription, α is defined as a continuous parameter, 0≤α≤1, and each of its values corresponds with a different discretization rule for the stochastic differential equation. α=0 corresponds to the Itô interpretation, while α=1/2 corresponds to the Stratonovich one. Additionally, α=1 is known as the Hänggi–Klimontovich (or kinetic) interpretation [6,26]. It is always good to emphasize that each stochastic prescription leads to a different stochastic evolution of Equation (3) and determines specific rules of differential calculus. Thus, the discretization prescription is an inherent component of the model.

To achieve thermodynamic equilibrium, the drift force f(x) must be connected to the potential U(x) and the dissipation function g(x) via a generalized Einstein relation [19], in such a way that

(5) $$f\left(x\right)=-\frac{1}{2}g^{2}\left(x\right)\frac{dU(x)}{dx}.$$

Under this condition, the Langevin Equation (3) can be rewritten as

(6) $$\frac{dx}{dt}\overset{\alpha }{=}-\frac{1}{2}g^{2}\left(x\right)\frac{dU(x)}{dx}+g\left(x\right)\eta \left(t\right).$$

The equilibrium probability distribution over long times takes the form

(7) $$P_{\mathrm{eq}}\left(x\right)=N\;e^{-\frac{1}{\sigma^{2}}U_{\mathrm{eq}}\left(x\right)},$$

where N is a normalization constant, and the equilibrium potential $U_{\mathrm{eq}}\left(x\right)$, obtained by solving the related stationary Fokker–Planck equation [19], is represented by

(8) $$U_{\mathrm{eq}}\left(x\right)=U\left(x\right)+\left(1-\alpha \right)\sigma^{2}\ln\left(\mu g^{2}\left(x\right)\right),$$

where μ is an arbitrary scale parameter. Naturally, no physical observable can depend on this parameter, as it merely represents a constant shift in the potential energy reference. Notice that the equilibrium distribution depends on the original potential as well as on the diffusion function g(x) and the stochastic prescription α. Notably, α=1 (the kinetic interpretation) is the unique stochastic prescription which results in the Boltzmann distribution, $U_{\mathrm{eq}}\left(x\right)=U\left(x\right)$.

There is a relation between stochastic differential equations defined in different prescriptions. Sometimes, it could be useful to represent the same stochastic process in a different prescription. In this context, the process given by Equation (3) in the α prescription can be formulated by using an alternative differential equation defined in another discretization (say, in the stochastic prescription β) through the following equation:

(9) $$\frac{dx(t)}{dt}\overset{\beta }{=}F_{\beta }\left(x\left(t\right)\right)+g\left(x\left(t\right)\right)\eta \left(t\right),$$

with

(10) $$F_{\beta }\left(x\right)=f\left(x\right)+\left(\alpha -\beta \right)\sigma^{2}g\left(x\right)g^{\prime }\left(x\right)\;,$$

where primes, ${\left(\;\right)}^{\prime }$, means derivative with respect to *x*. A thorough proof is provided in the appendix of ref. [3]. This property is very useful because, depending on the calculation techniques, some prescriptions are more convenient or easier to be implemented. For instance, for analytic calculations, the Stratonovich prescription β=1/2 is simpler because the calculus rules, such as the chain rule or integration by parts, turn out to be the usual ones. Alternatively, the Itô interpretation (β=0) is the unique prescription which guarantees the non-anticipating property for stochastic calculus [4] and makes easier to perform numerical simulations.

Although the techniques and results presented here are general, we illustrate the equilibrium potential $U_{\mathrm{eq}}\left(x\right)$ using a simple bistable model,

(11) $$U\left(x\right)=\frac{1}{4}x^{4}-\frac{1}{2}x^{2}+px,$$

in which the term px breaks the parity symmetry. For the diffusion function, we choose

(12) $$g\left(x\right)=1+\lambda x^{2},$$

where λ quantifies the multiplicative nature of the noise. For λ=0, the noise is additive. To outline the general characteristics of the equilibrium potential, we express Equations (11) and (12) in arbitrary units. A discussion about dimensions will follow the presentation of our main result, Equation (35).

**Figure 1:**
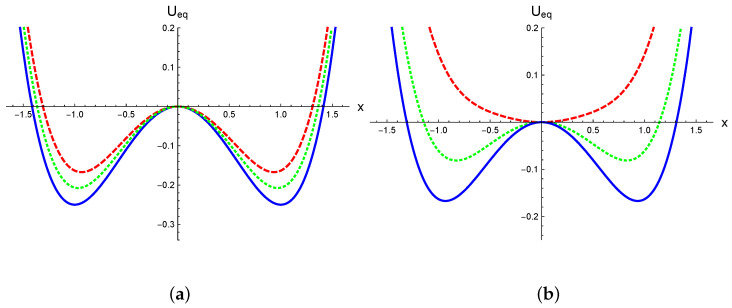
The equilibrium potential $U_{\mathrm{eq}}\left(x\right)$, as defined in Equation (8) with p=0, is shown. In panel (**a**), $\sigma^{2}=0.095$ is fixed. The solid line corresponds to the anti-Itô prescription (α=1), the dotted line represents the Stratonovich prescription (α=1/2), and the dashed line depicts the Itô interpretation (α=0). In panel (**b**), all curves are calculated using the Itô interpretation. The solid line corresponds to $\sigma^{2}=0.055$, the dotted line to $\sigma^{2}=0.12$, and the dashed line to $\sigma^{2}=0.25$. In both panels, we set λ=1.2.

**Figure 2:**
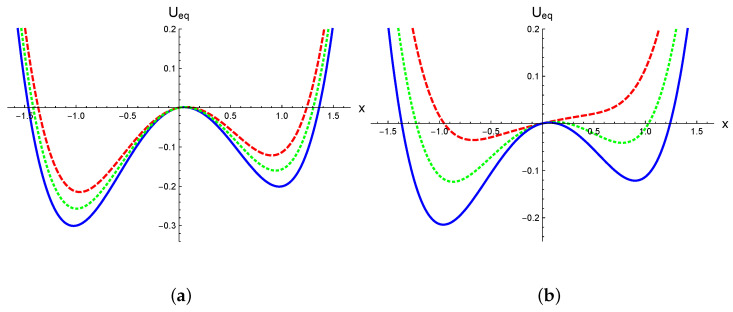
The equilibrium potential $U_{\mathrm{eq}}\left(x\right)$, given by Equation (8), with p≠0. In panel (**a**), σ=0.5 is fixed. The solid line corresponds to the anti-Itô prescription (α=1), the dotted line represents the Stratonovich prescription (α=1/2), and the dashed line depicts the Itô interpretation (α=0). In panel (**b**), all curves are calculated using the Itô interpretation. The solid line corresponds to $\sigma^{2}=0.055$, the dotted line to $\sigma^{2}=0.12$, and the dashed line to $\sigma^{2}=0.2$. In both panels, we set λ=1.2 and p=0.05.

The potential U(x) exhibits two minima (degenerated for p=0) and a local maximum. The multiplicative noise introduces significant effects in the equilibrium potential. In Figure 1, the equilibrium potential $U_{\mathrm{eq}}\left(x\right)$, defined by Equation (8), is illustrated for the simplest symmetric model described by Equations (11) and (12) (with p=0), considering different values of the parameters σ and α. Figure 1a shows the equilibrium potential for $\sigma^{2}=0.095$ under various stochastic prescriptions, α=0,1/2,1. For α=1, we find $U_{\mathrm{eq}}\left(x\right)=U\left(x\right)$, with the minima fixed at $x_{\min}=\pm 1$. In contrast, under the Stratonovich and Itô prescriptions, the minima shift toward the origin. In Figure 1b, the curves are computed using the Itô prescription for different noise intensities, $\sigma^{2}=0.055,0.12,0.25$. In this case, as the noise increases, the minima move closer to zero; for the largest value $\sigma^{2}=0.25$, the equilibrium potential exhibits a single global minimum at $x_{\min}=0$. As noted in ref. [3], this behavior resembles a second-order phase transition induced by spontaneous symmetry breaking. This is in contrast to the asymmetric case shown in Figure 2, where we fix p=0.05. In this case, there is a metastable local minimum and a global minimum separated by a barrier. Similarly to the symmetric case, in Figure 2a, we show how the minima approach as the prescription goes from α=1 to α=0, as well as the differences in the height of the barrier for the stochastic prescriptions. This aspect clearly impacts the validity range of the Kramers rate. In Figure 2b, we show the equilibrium potential in the Itô prescription for different values of noise. It is possible to see that with increasing noise, the barrier height decreases and the metastable state disappears, in a similar way to a spinodal decomposition mechanism. Therefore, the effects of multiplicative noise on the equilibrium properties of the systems are nontrivial, in the symmetric as well as in the asymmetric case.

## 3. Path Integral Representation of Langevin Dynamics with Multiplicative White Noise

We present here the formalism used to compute conditional probabilities. This section is not entirely novel; however, it is necessary to introduce the notation and to make a self-contained presentation. We briefly outline the method and refer the interested reader to the cited references for technical details.

Conditional probabilities are expressed by the means of the path integral representation of the generating functional of stochastic correlations. In order to build the path integral, it is convenient to work in the Stratonovich prescription. Thus, using Equation (9), with β=1/2, the stochastic process given by Equation (6), defined in the α-prescription sense, is described by the Langevin equation

(13) $$\frac{dx(t)}{dt}\overset{S}{=}F_{S}\left(x\left(t\right)\right)+g\left(x\left(t\right)\right)\eta \left(t\right),$$

with

(14) $$F_{S}\left(x\right)=f\left(x\right)+\left(\frac{2\alpha -1}{2}\right)\sigma^{2}g\left(x\right)g^{\prime }\left(x\right)$$

where, now, the stochastic differential equation is integrated in the Stratonovich sense. In this equation, α is just a parameter contained in the definition of the drift force $F_{S}$.

The transition probability $P(x_{f},t_{f}|x_{i},t_{i})$ plays a fundamental role in studying any dynamical property of a stochastic process. It represents the conditional probability of the system being in the state $x_{f}$ at the time $t_{f}$, given that it was in the state $x_{i}$ at the time $t_{i}$. Within the path integral formalism, this probability can be expressed as [21,23,27]

(15) $$P\left(x_{f},t_{f}|x_{i},t_{i}\right)=\int Dx\;\det^{-1}\left(g\right)\;e^{-\frac{1}{\sigma^{2}}S\left[x\right]}\;,$$

where the “action” S[x] is given by

(16) $$S\left[x\right]=\int_{t_{i}}^{t_{f}}dt\left\{\frac{1}{2g^{2}}{\left[\frac{dx}{dt}-F_{S}+\frac{1}{2}\sigma^{2}gg^{\prime }\right]}^{2}+\frac{\sigma^{2}}{2}F_{S}^{\prime }-\frac{\sigma^{4}}{8}g^{\prime 2}\right\},$$

with the boundary conditions $x\left(t_{i}\right)=x_{i}$ and $x\left(t_{f}\right)=x_{f}$. Equations (15) and (16) coincide with the Onsager–Mashlup representation [28] of the conditional probability for the stochastic process governed by Equation (13). Although slightly different versions of Equation (16) have been reported [21,23,27], essentially due to the covariant properties of the formalism, the differences are of the order $O\left(\sigma^{4}\right)$ [29] and do not affect the present computation of the transition probability, as we explain below.

Rewriting the action in an alternative form is very insightful. By expanding the squared bracket in Equation (16) and applying Equations (14), (5), and (8), followed by integration by parts, we obtain

(17) $$S\left[x\right]=\frac{\Delta U_{\mathrm{eq}}}{2}+\int_{t_{i}}^{t_{f}}dt\;L\left(x,\dot{x}\right)\;,$$

where $\dot{x}$ denotes the time derivative. The first term is a state function defined by the equilibrium potential $U_{\mathrm{eq}}$, evaluated in the system’s initial and final states, such that $\Delta U_{\mathrm{eq}}=U_{\mathrm{eq}}\left(x_{f}\right)-U_{\mathrm{eq}}\left(x_{i}\right)$, where $U_{\mathrm{eq}}$ is determined by Equation (8). The Lagrangian can be expressed in the following suggestive form:

(18) $$L=\frac{1}{2}\left(\frac{1}{g^{2}\left(x\right)}\right){\dot{x}}^{2}+V\left(x\right)\;,$$

where

(19) $$V\left(x\right)=\frac{g^{2}}{2}\left[{\left(\frac{U_{\mathrm{eq}}^{\prime }}{2}\right)}^{2}-\sigma^{2}\left(\frac{U_{\mathrm{eq}}^{\prime \prime }}{2}+\frac{g^{\prime }}{g}U_{\mathrm{eq}}^{\prime }\right)\right]+O\left(\sigma^{4}\right).$$

The first interesting result is that, for the order $\sigma^{2}$, all the dependence on the parameter α is encoded in the equilibrium potential $U_{\mathrm{eq}}$ (Equation (8)), strengthening the role of this quantity not only for equilibrium properties but also for dynamic evolution.

Replacing Equation (17) in Equation (15), the conditional probability takes the form

(20) $$P\left(x_{f},t_{f}|x_{i},t_{i}\right)=e^{-\frac{\Delta U_{\mathrm{eq}}}{2\sigma^{2}}}K\left(x_{f},t_{f}|x_{i},t_{i}\right)$$

with $K(x_{f},t_{f}|x_{i},t_{i})$ represented by

(21) $$K\left(x_{f},t_{f}|x_{i},t_{i}\right)=\int \left[Dx\right]\;e^{-\frac{1}{\sigma^{2}}\int_{t_{i}}^{t_{f}}dt\;L\left(x,\dot{x}\right)}\;,$$

which is commonly known as a *propagator* in the quantum mechanics literature. Here, the initial and final conditions are considered $x_{i}=x\left(t_{0}\right)$ and $x_{f}=x\left(t_{N}\right).$ The functional integration measure takes the form

(22) $$\left[Dx\right]=Dx\;\det^{-1}g=\lim_{\begin{array}{c} N\to \infty \\ \Delta t\to 0 \end{array}}\prod_{n=0}^{N-1}\frac{dx_{n}}{\sqrt{\Delta t\;g^{2}\left(\frac{x_{n}+x_{n+1}}{2}\right)}}$$

with $x_{n}=x\left(t_{n}\right)$.

Notably, the Equation (21) is the exact propagator for a quantum particle with a position-dependent mass, $m\left(x\right)=1/g^{2}\left(x\right)$, moving in a potential, V(x), written within the imaginary time path integral formalism t→−it [23]. In this context, the noise intensity $\sigma^{2}$ serves as the counterpart of *ℏ* in the quantum theory.

## 4. Weak Noise Expansion and the Kramers Escape Rate

We analytically compute $K(x_{f},t_{f}|x_{i},t_{i})$ in Equation (21) in a weak noise approximation. The propagator can be written in the saddle-point expansion with Gaussian fluctuations as

(23) $$K\left(x_{f},t_{f}|x_{i},t_{i}\right)=\sum_{n}e^{-\frac{1}{\sigma^{2}}S\left[x_{cl}^{\left(n\right)}\right]}\int \left[D\delta x_{n}\right]\;e^{-\frac{1}{2}\int dtdt^{\prime }\;\delta x_{n}\left(t\right)S^{\left(2\right)}\left[x_{cl}^{\left(n\right)}\right]\left(t,t^{\prime }\right)\delta x_{n}\left(t^{\prime }\right)}\;.$$

The functions $x_{cl}^{\left(n\right)}\left(t\right)$, with n=1,2,3,…, denote the solutions (or approximate solutions) of the equation of motion

(24) $${\left.\frac{\delta S[x]}{\delta x(t)}\right|}_{x=x_{cl}^{\left(n\right)}}=\frac{d^{2}x_{cl}^{\left(n\right)}}{dt^{2}}-g^{2}V^{\prime }-\frac{g^{\prime }}{g}{\left({\dot{x}}_{cl}^{\left(n\right)}\right)}^{2}=0$$

with $x_{cl}^{\left(n\right)}\left(t_{i}\right)=x_{i}$ and $x_{cl}^{\left(n\right)}\left(t_{f}\right)=x_{f}$ as initial and final conditions.

The classical action in Equation (23) is

(25) $$S\left[x_{cl}^{\left(n\right)}\right]=\int_{t_{i}}^{t_{f}}dt\;L\left(x_{cl}^{\left(n\right)}\left(t\right),{\dot{x}}_{cl}^{\left(n\right)}\left(t\right)\right)$$

while the fluctuation kernel is given by

(26) $$\begin{array}{cc} S_{cl}^{\left(2\right)}\left(t,t^{\prime }\right) & ={\left.\frac{\delta^{2}S\left[x\right]}{\delta x\left(t^{\prime }\right)\delta x\left(t\right)}\right|}_{x\left(t\right)=x_{cl}^{\left(n\right)}\left(t\right)}. \end{array}$$

In Equation (23), the functional integration measure is represented as

(27) $$\left[D\delta x_{n}\right]=\lim_{\begin{array}{c} N\to \infty \\ \Delta t\to 0 \end{array}}\prod_{j=0}^{N-1}\frac{d\delta x_{j}^{\left(n\right)}}{\sqrt{\Delta t\;g^{2}\left(\frac{x_{cl}^{\left(n\right)}\left(t_{j}\right)+x_{cl}^{\left(n\right)}\left(t_{j+1}\right)}{2}\right)}}$$

and fluctuations satisfy the boundary conditions $\delta x_{n}\left(t_{i}\right)=\delta x_{n}\left(t_{f}\right)=0$.

**Figure 3:**
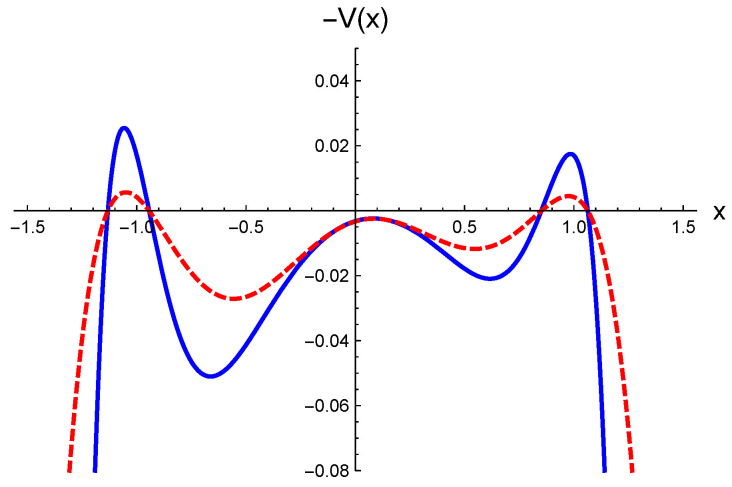
The opposite of the potential V(x) given by Equation (19), for the Itô prescription (α=0), with σ=0.1 and p=0.08. The additive noise case, given by λ=0 in the diffusion function (Equation (12)), is depicted with dashed lines, while multiplicative noise, taking λ=1, is illustrated with a continuous line.

The main task is to compute all solutions of Equation (24) and the corresponding fluctuation integral around each one. In Figure 3, the opposite of the potential V(x) is displayed, considering its general form given by Equation (19). The first observation is that −V(x) has three maxima and two minima. The positions of the lateral maxima roughly align with the minima of the potential U(x), with a difference of the order $\sigma^{2}$. The primary effect of the diffusion function is an increase in the curvature at each maximum by a factor proportional to $g^{2}\left(x_{\max}\right)>1$. A key feature, important for computing conditional probabilities, is that the height differences between the peaks are of the order $\sigma^{2}$. Therefore, in a weak noise regime, the three maxima are quasi-degenerated.

For simplicity, let us generically call x=a the position of one of the lateral maxima, say, x∼−1 in Figure 3, and x=b the position of the other maximum x∼1 in the same figure. Moreover, we call $x=x_{m}$ the position of the central maximum (x∼0 in the figure).

We are interested in computing, for example, the probability that the system remains within one of the wells of U(x), specifically around x=a. In this case, given that $x_{i}=x_{f}$, $\Delta U_{eq}=0$ in Equation (20), and the propagator corresponds to the probability of the system staying in the same state. Thus, we want to compute

(28) P(a,t/2|a,−t/2)=K(a,t/2|a,−t/2).

The first task is to compute solutions of the equation of motion, Equation (24), with the conditions x(±t/2)=a. A trivial solution is simply $x_{cl}=a$, and it is possible to compute its contribution to the propagator. Details of the calculation can be found in ref. [3]. So, we obtain

(29) $$K^{\left(0\right)}\left(a,t/2|a,-t/2\right)={\left(\frac{g_{a}^{2}U_{\mathrm{eq}}^{\prime \prime }\left(a\right)}{2\pi \sigma^{2}}\right)}^{1/2}\;,$$

denoting g(a) by $g_{a}$ and where the superscript (0) is used to highlight the contribution of the constant solution to the propagator.

Moreover, for very long times, the dynamics of the system exhibit topological time-dependent solutions with finite action that connect the maxima of the potential. These solutions, commonly referred to as instantons and anti-instantons, play a critical role in calculating the propagator. Over extended time intervals, nontrivial contributions to the path integral arise from the well-separated superposition of instantons and anti-instantons. The methodology for summing over these configurations, known as the dilute instanton/anti-instanton gas approximation, has been extensively developed to compute tunneling amplitudes in quantum mechanics [30,31,32]. In the context of stochastic processes, this approach has been applied to systems driven by additive white noise [33], as well as to scenarios involving colored noise [34,35,36,37]. Stochastic systems with multiplicative noise and symmetric potentials were recently treated in ref. [3]. In this paper, we generalize the results presented in the last reference to asymmetric potentials.

In our case, the simplest instanton/anti-instanton solution that satisfies the boundary conditions is a trajectory that begins at x=a at the time −t/2, goes to $x=x_{m}$ at some intermediate time, $t_{m}$, and goes back to x=a at the time t/2. We use the schematic notation $a⇆x_{m}$ to indicate this trajectory. By following standard, albeit laborious, procedures, we obtain [3]

(30) $$K^{\left(1\right)}\left(a,\frac{t}{2}|a,-\frac{t}{2}\right)=-g^{2}\left(x_{m}\right)t\;K^{\left(0\right)}\;\Gamma_{a}\;,$$

where $K^{\left(0\right)}$ represents the contribution from the constant solution, as expressed in Equation (29), and

(31) $$\Gamma_{a}=\frac{{\left(U_{\mathrm{eq}}^{\prime \prime }\left(a\right)\left|U_{\mathrm{eq}}^{\prime \prime }\left(x_{m}\right)\right|\right)}^{1/2}}{2\pi }\exp\left\{-\frac{U_{\mathrm{eq}}\left(x_{m}\right)-U_{\mathrm{eq}}\left(a\right)}{\sigma^{2}}\right\}\;.$$

As can be observed in Equation (30), at long times, the contribution to the propagator of a simple instanton/anti-instanton configuration is a linear function of time. Furthermore, the structure of the coefficient $\Gamma_{a}$ is revealing. This is the unique term in the theory containing the equilibrium potential, $U_{\mathrm{eq}}$, which, at the same time, completely encloses the information relative to the stochastic calculus. Additionally, $\Gamma_{a}$ depends on the curvature at the maximum and the minimum of the equilibrium potential, $U_{\mathrm{eq}}^{\prime \prime }\left(x_{m}\right)$ and $U_{\mathrm{eq}}^{\prime \prime }\left(a\right)$, respectively, and on the barrier height, $U_{\mathrm{eq}}\left(x_{m}\right)-U_{\mathrm{eq}}\left(a\right)$, without any dependence on the explicit details of $U_{\mathrm{eq}}\left(x\right)$.

The propagator also receives important contributions from other solutions of Equation (24). There are trajectories that contain two instantons and two anti-instantons, which we schematically depict as a⇆b. This is the case, for example, of trajectories beginning in x=a that go to x=b, passing through $x=x_{m}$, and then return to x=a. The contribution of these trajectories to the propagator can be computed by following the same technique as for the previous calculation of $a⇆x_{m}$ trajectories [3]. Performing so, in this case, we find

(32) $$K^{\left(2\right)}\left(a,\frac{t}{2}|a,-\frac{t}{2}\right)=\frac{{\left(g^{2}\left(x_{m}\right)t\right)}^{2}}{2!}\;K^{\left(0\right)}\;\Gamma_{a}\Gamma_{b}\;,$$

where $\Gamma_{b}$ has the same structure of $\Gamma_{a}$ given by Equation (31), just by changing a→b in all places. As can be seen, trajectories of this kind (a⇆b) produce a quadratic time contribution to the propagator, with a coefficient proportional to $\Gamma_{a}\Gamma_{b}$.

Combinations of these types of configurations are, in the long-time approximation, also quasi-solutions of Equation (24) and should be considered in the summation of the propagator of Equation (23). Typical trajectories would be

(33) $$a\overset{p\;\mathrm{times}}{⇆}x_{m}\overset{q\;\mathrm{times}}{⇆}b$$

where p,q are integer numbers. The final infinite summation is a combinatory problem that can be exactly summed [3,33], obtaining

(34) $$P\left(a,t/2|a,-t/2\right)=\frac{K^{\left(0\right)}}{\Gamma_{a}+\Gamma_{b}}\left\{\Gamma_{b}+\Gamma_{a}e^{-rt}\right\}$$

where the decay rate is given by $r=g{\left(x_{m}\right)}^{2}\left(\Gamma_{a}+\Gamma_{b}\right)/2$. Explicitly, we find, for the decay rate,

(35) $$r=\frac{g{\left(x_{m}\right)}^{2}}{2}\left(\frac{\sqrt{{\tilde{\omega }}_{a}\left|{\tilde{\omega }}_{m}\right|}}{2\pi }e^{-\frac{\Delta U_{\mathrm{eq}}^{a}}{\sigma^{2}}}+\frac{\sqrt{{\tilde{\omega }}_{b}\left|{\tilde{\omega }}_{m}\right|}}{2\pi }e^{-\frac{\Delta U_{\mathrm{eq}}^{b}}{\sigma^{2}}}\right)$$

where ${\tilde{\omega }}_{a}=U_{\mathrm{eq}}^{\prime \prime }\left(a\right)$, ${\tilde{\omega }}_{b}=U_{\mathrm{eq}}^{\prime \prime }\left(b\right)$ and ${\tilde{\omega }}_{m}=U_{\mathrm{eq}}^{\prime \prime }\left(x_{m}\right)\;$ are the local curvatures at the minima and the maximum of the equilibrium potential. Moreover,

(36) $$\begin{array}{c} \Delta U_{\mathrm{eq}}^{a}=U_{\mathrm{eq}}\left(x_{m}\right)-U_{\mathrm{eq}}\left(x_{a}\right) \end{array}$$

(37) $$\begin{array}{c} \Delta U_{\mathrm{eq}}^{b}=U_{\mathrm{eq}}\left(x_{m}\right)-U_{\mathrm{eq}}\left(x_{b}\right) \end{array}$$

are the barrier height, measured from each of the asymmetric minima.

Equation (35) is the main contribution of this paper. It presents an analytic expression for the Kramers decay rate in the case of state-dependent diffusion for asymmetric potentials. The main approximations involved in our calculation are the assumption of a weak noise, $\sigma^{2}\ll \Delta U_{\mathrm{eq}}^{a}∼\Delta U_{\mathrm{eq}}^{b}$, and the expansion for long times, rt≫1.

At this point, it is useful to verify the dimensional consistency of Equation (35). Starting from the overdamped Langevin equation, Equation (3), and using the Einstein relation, Equation (5), we deduce that $\left[g\right]=L/(\left[\sigma \right]\sqrt{\left[t\right]})$ and $\left[U\right]=\left[\sigma^{2}\right]$, where [·] denotes the dimensional units of a quantity and *L* represents a characteristic unit of length. From these considerations, it follows straightforwardly that [r]=1/[t], as expected. Interestingly, given a characteristic length scale, [x]=L, and the energy scale $\left[\sigma^{2}\right]$, the characteristic time scale is determined by the value of the diffusion function $g^{2}$. These are the only parameters that appear in the Langevin equation, Equation (3).

Equation (35) is a nontrivial generalization of previous results. For instance, for p=0 in the original potential, $U_{\mathrm{eq}}\left(x\right)=U_{\mathrm{eq}}\left(-x\right)$ and, in this case, a=−b. In these conditions, Equation (35) reduces to the expression computed in ref. [3]. On the other hand, for λ=0 or g(x)=1, the stochastic dynamics are additive and our results converge to that of reference [33].

## 5. Numerical Simulations

For the purpose of validating the expression for the Kramers escape rate given by Equation (35), we performed extensive numerical simulations for the stochastic process governed by the Langevin Equation (6) by using the Euler–Maruyama scheme. This algorithm was based on an Itô interpretation of the stochastic differential equation (SDE). Therefore, given that the Langevin equation was initially considered in the general α-prescription, it had to be transformed to an Itô-defined Langevin equation by the means of Equation (10), taking β=0. Consequently, any α-defined SDE, for 0≤α≤1, could be represented through the following Itô differential equation:

(38) $$\frac{dx}{dt}=-\frac{1}{2}g^{2}\left(x\right)\frac{dU(x)}{dx}+\sigma^{2}\alpha g\left(x\right)g^{\prime }\left(x\right)+g\left(x\right)\eta \left(t\right).$$

For the model described by Equations (11) and (12), the Itô stochastic differential equation can be explicitly written as

(39) $$dx=\frac{\left(1+\lambda x^{2}\right)}{2}\left\{\left(1+\lambda x^{2}\right)\left[x\left(1-x^{2}\right)-p\right]+4\lambda \sigma^{2}\alpha x\right\}dt+\left(1+\lambda x^{2}\right)dW\;,$$

where W(t) is a standard Wiener process with 〈W(t)〉=0 and $\langle W\left(t\right)W\left(t^{\prime }\right)\rangle =\sigma^{2}\min\left(t,t^{\prime }\right)$. The white noise η(t) is formally defined as the time derivative of the Wiener process. This relation is understood in the context of distributions, where η(t) represents a generalized derivative [4].

**Figure 4:**
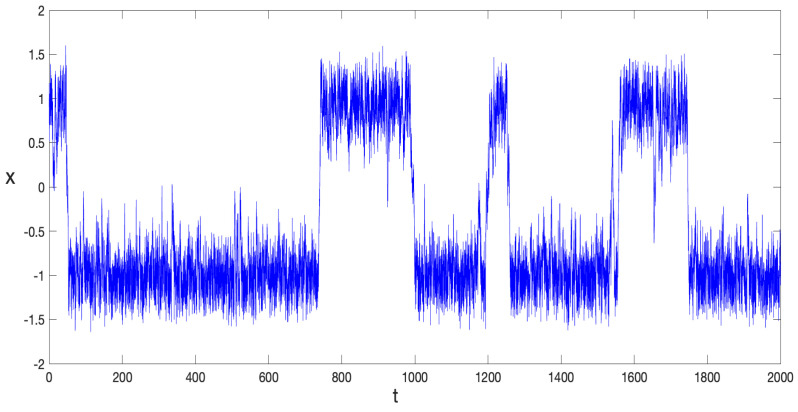
x(t), obtained by numerically integrating Equation (39) for a specific noise realization, with the parameters p=0.08; λ=0.5; α=1; and $\sigma^{2}=0.085$. The time interval 0<t<2000 was divided into $7\times 10^{4}$ steps.

In Figure 4, a typical path from Equation (39) is depicted for a particular noise realization and by making p=0.08 in the expression of the potential function U(x), Equation (11). With the initial condition x(0)=1 fixed, the dynamics of the stochastic variable x(t) become evident, as they fluctuate around the potential minima $x_{\min}∼\pm 1$, transitioning between them at seemingly irregular intervals. In this case, the global minimum of the potential is the negative one, $x_{\min}∼-1$, and its barrier is higher, further increasing the likelihood that the system remains around this minimum for longer periods.

**Figure 5:**
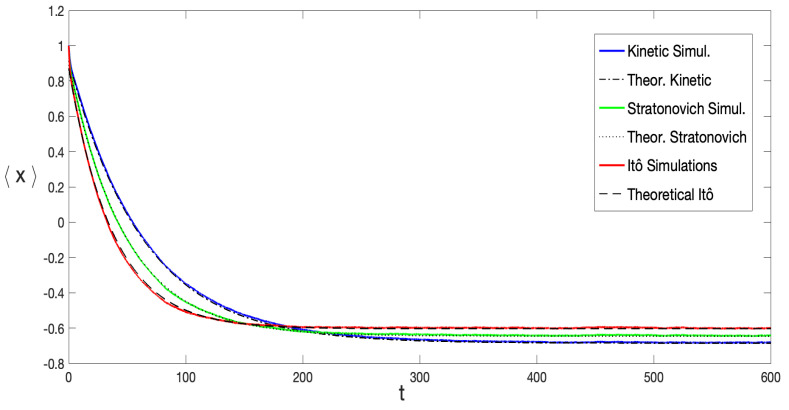
〈x(t)〉 computed from Equation (39), obtained by averaging over $8\times 10^{4}$ noise configurations. The initial condition x(0)=1 and parameter values λ=0.5 and $\sigma^{2}=0.085$ were fixed. The curves represent different stochastic prescriptions: α=0; α=1/2; and α=1. Solid lines represent the numerical simulations while the dashed, dotted, and dash–dotted lines correspond to the theoretical expression given by Equation (41), in the Itô, Stratonovich, and Kinetic prescriptions, respectively. The only fitting parameter was the amplitude A∼1.

To ensure reliable statistical results, the mean value 〈x(t)〉 was computed over a significant number of distinct noise realizations. Figure 5 displays the results obtained by averaging over $8\times 10^{4}$ noise configurations for various stochastic prescriptions. As expected, 〈x(t)〉 converges exponentially toward the equilibrium value

(40) $$x_{\mathrm{eq}}\equiv {\langle x\rangle }_{\mathrm{eq}}=\int_{-\infty }^{+\infty }dx\;x\;P_{\mathrm{eq}}\left(x\right)$$

with the equilibrium probability distribution $P_{\mathrm{eq}}\left(x\right)$ represented by Equation (7).

**Figure 6:**
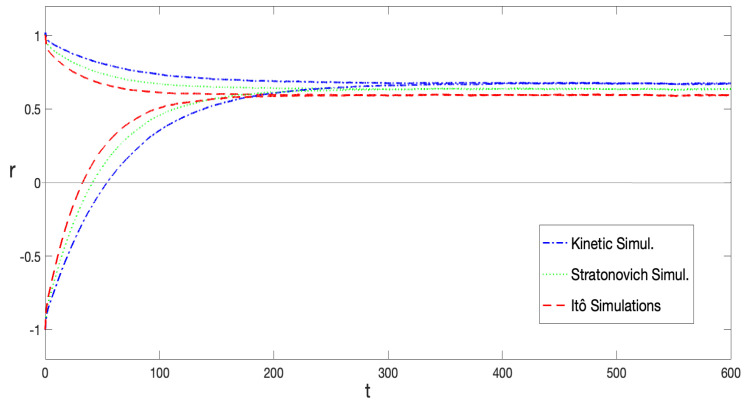
〈x(t)〉 computed from Equation (39), obtained by averaging over $8\times 10^{4}$ noise configurations for two different initial conditions, x(0)=1 and x(0)=−1. Numerical simulations were performed for different stochastic prescriptions: α=0; α=1/2; and α=1. The parameter values λ=0.5 and $\sigma^{2}=0.085$ were fixed. The values of *r* and $x_{\mathrm{eq}}$ were independent of the initial conditions.

Figure 5 also reveals that the typical decay rate varied across different stochastic prescriptions, with $r_{I}>r_{S}>r_{K}$, where $r_{I}$, $r_{S}$, and $r_{K}$ represent the decay rates for the Itô, Stratonovich, and Kinetic prescriptions, respectively. This is aligned with the observation in Figure 2a, which shows that the height of the equilibrium potential barrier increases as α grows. This behavior is independent of the initial condition used in the SDE integration. As shown in Figure 6, simulations starting near both the metastable and global minima illustrate this point. It is possible to observe that the reached equilibrium state as well as the decay rates do not depend on initial conditions. It can also be distinguished that the equilibrium state for the Itô prescription has the smallest absolute value, while it is the largest for the anti-Itô convention. This is further represented in Figure 1a and Figure 2a; the Itô-defined SDE always exhibits the lowest potential barrier, facilitating transitions between minima in this prescription.

By means of the asymptotic conditional probability distribution derived in Section 4, Equation (34), it was possible to obtain an analytical expression for 〈x(t)〉 in the long-time limit. It can be shown that, for $t\gg r^{-1}$,

(41) $$\langle x\left(t\right)\rangle =A\;e^{-rt}+x_{\mathrm{eq}},$$

where *r* is given by Equation (35) and *A* is a constant used as a fitting parameter. Equation (41) was used to fit the numerical simulations curves and is graphically represented in Figure 5, demonstrating excellent agreement between the theoretical result and simulations across all three stochastic prescriptions.

As an additional validation of the results, we plotted the logarithm of fluctuations around the equilibrium state,

(42) $$\ln\left[\delta x(t)\right]=-rt+\ln A\;,$$

where $\ln\left[\delta x(t)\right]=\ln\left[\langle x\left(t\right)\rangle -x_{\mathrm{eq}}\right]$, and a linear least-square fitting of $\ln\left[\delta x(t)\right]$ was made. We used an arbitrary scale to split up $\ln\left[\delta x/A\right]$, since it does not affect the value of rt. Notice that the decay rate is given by the slope of the linear function and does not depend on any fitting parameter. This number, measured from the data, must be compared with the analytic expression of the decay rate given by Equation (35). Using this approach, we explored a broad range of the parameter space $\left\{\alpha ,\sigma^{2}\right\}$.

**Figure 7:**
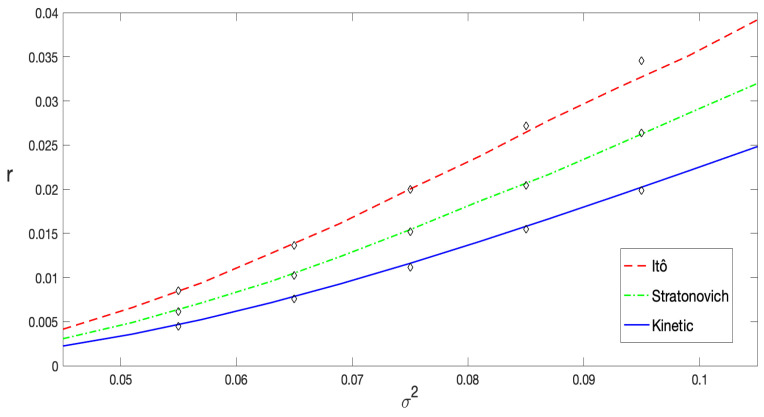
The decay rate *r* computed through Equation (35), represented as a function of the noise intensity $\sigma^{2}$. The solid line represents the decay rate for the Itô prescription, while the dashed and dash–dotted curves correspond to the Stratonovich and kinetic (or anti-Itô) interpretations, respectively. The points (diamonds) were obtained via the least-squares fitting of $\ln\left[\langle x\left(t\right)\rangle -x_{\mathrm{eq}}\right]$ based on numerical simulations for each case. For all cases, the parameters were fixed at λ=0.5 and p=0.08.

**Figure 8:**
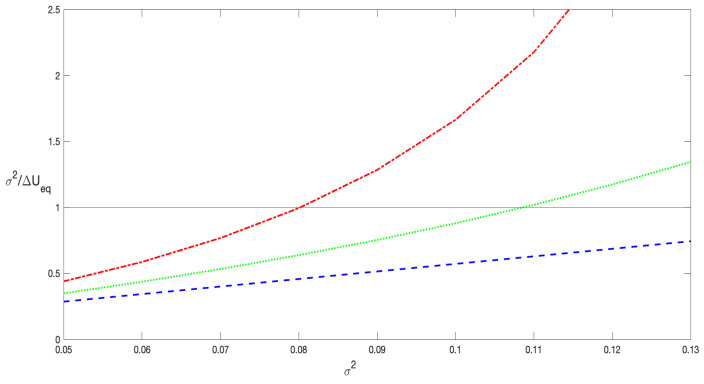
$\sigma^{2}/\Delta U_{\mathrm{eq}}$ as a function of the noise $\sigma^{2}$. The dashed line represents the kinetic prescription α=1, the dotted line corresponds to the Stratonovich prescription, α=1/2, while the Itô prescription, α=0, is represented by the dash–dotted line. The parameters λ=0.5 and p=0.08 were fixed for all the curves.

In Figure 7, the decay rate *r*, represented as a function of the noise intensity $\sigma^{2}$, is displayed for the three stochastic prescriptions under consideration. The continuous, dashed, and dash–dotted curves were derived from Equation (35), corresponding to the Itô, Stratonovich, and kinetic (or anti-Itô) interpretations, respectively. Numerical results, obtained via the least-squares fitting of $\ln\left[\delta x(t)\right]$, are represented by diamond markers. Each diamond reflects computations based on at least $8\times 10^{4}$ numerical simulations for each pair of values of α and $\sigma^{2}$, with λ=0.5 and p=0.08. A remarkable agreement can be perceived across nearly the entire range of noise intensity. A slight deviation occurs for higher noise values, where $\Delta U_{\mathrm{eq}}/\sigma^{2}∼1$, as expected, since the Arrhenius approximation becomes less reliable in this regime. The first noticeable deviations in the results correspond to the Itô interpretation, as further highlighted in Figure 8.

**Figure 9:**
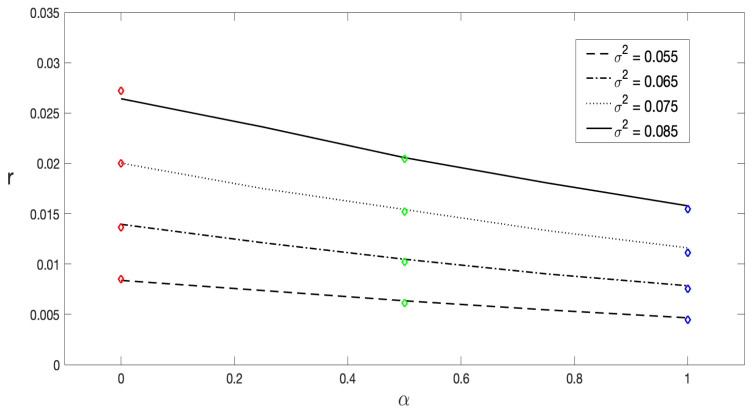
The decay rate *r* computed through Equation (35) for different values of $\sigma^{2}$, represented as a function of the stochastic prescription α. The solid line corresponds to $\sigma^{2}=0.085$ and the dashed one corresponds to $\sigma^{2}=0.075$, while the dash–dotted and dotted lines correspond to $\sigma^{2}=0.065$ and $\sigma^{2}=0.055$, respectively. The points (diamonds) were obtained via the least-squares fitting of $\ln\left[\langle x\left(t\right)\rangle -x_{\mathrm{eq}}\right]$ based on numerical simulations for each case. The parameters λ=0.5 and p=0.08 were fixed for all the curves.

The decay rate *r* as a function of the stochastic prescription α, with 0≤α≤1, is shown in Figure 9, for a noise range from $\sigma^{2}=0.055$ to $\sigma^{2}=0.085$. Once again, an excellent agreement between the theoretical predictions derived from Equation (35) and the results obtained through numerical simulations of the Langevin equation is observed. It is important to highlight that this analytic expression was obtained in the limit $\sigma^{2}\ll \Delta U_{\mathrm{eq}}=U_{\mathrm{eq}}\left(x_{max}\right)-U_{\mathrm{eq}}\left(x_{min}\right)$. Therefore, the most precise and accurate values of $r(\sigma^{2})$, given by Equation (35) are expected for $\sigma^{2}/\Delta U_{\mathrm{eq}}\ll 1$.

For the sake of comparison between the different stochastic prescriptions, in Figure 8, $\sigma^{2}/\Delta U_{\mathrm{eq}}$ is displayed as a function of the noise $\sigma^{2}$ for the Itô (dash–dotted line), Stratonovich (dotted line), and kinetic (dashed line) conventions. The figure shows that in the kinetic prescription, this quantity is always less than unity within the studied noise range. This indicates that the noise intensity is consistently much lower than the potential barrier, resulting in an excellent agreement, as observed in Figure 7 and Figure 9. On the other hand, for the Stratonovich and Itô prescriptions, we observe a critical value of $\sigma^{2}$ in which $\sigma^{2}/\Delta U_{\mathrm{eq}}∼1$. For noise intensity above this critical value, a good agreement between analytical and simulation results is no longer expected.

Most of the results exhibited in Figure 7 and Figure 9 correspond to values of $\sigma^{2}$ within the interval [0.055,0.085]. The results are highly accurate within this range; however, as pointed before, for the Itô prescription, the value of *r* at $\sigma^{2}=0.085$ slightly deviates from the numerical data. At this noise intensity, $\sigma^{2}/\Delta U_{\mathrm{eq}}∼1.2$. The deviation becomes more pronounced in Figure 7 for the Itô prescription at $\sigma^{2}=0.095$, as confirmed by the corresponding curve in Figure 8. This general behavior is expected because, as noted in Section 2, the barrier height decreases as α→0 and for increasing values of noise. Indeed, there is a critical noise level at which the barrier completely disappears and the system dramatically changes its dynamics.

## 6. Discussion and Conclusions

In this paper, we explored the dynamics of bistable systems influenced by multiplicative noise, presenting both analytical and numerical approaches to understand escape rates within asymmetric potentials. Our work generalized Kramers’s formula to systems with state-dependent diffusion, highlighting the impact of the stochastic prescription and asymmetry of the potential on equilibrium properties and transition rates. Among other applications, it serves as a starting point for studying stochastic resonance in systems governed by stochastic differential equations with multiplicative noise.

The main result of this paper is presented by Equation (35). The derived analytic expression for decay rates, validated through extensive numerical simulations, provides a robust framework for analyzing stochastic systems with multiplicative noise. Interestingly, the weak noise approximation was demonstrated to be effective, as evidenced by the excellent agreement between theoretical predictions and simulation data across various noise intensities and stochastic prescriptions.

In Figure 7, it can be clearly observed that the decay rate computed using the Itô prescription is greater than that computed with the Stratonovich interpretation, which in turn is greater than the rate computed with the Hänggi–Klimontovich prescription. Furthermore, in Figure 9, we demonstrate that the decay rate as a function of the prescription, r(α), is a monotonically decreasing function. This behavior is due to two main reasons. First, the decay rate is primarily related to the first moment of the probability distribution, as $rt∼\ln\left(\langle x(t)\rangle \right)$ in the long-time limit where rt≫1. It has been analytically shown [18,19], at least for short times, that in systems with state-dependent diffusion the stochastic prescription significantly influences the mean value 〈x(t)〉 while leaving the mean square displacement (MSD) $\langle x^{2}\left(t\right)\rangle$ relatively unchanged. This agrees with numerical simulations, which show that the scaling properties of the MSD are independent of the stochastic prescription. Indeed, the prescription only affects the prefactor of the MSD, with a rather weak dependence [15]. Second, the most significant effect of the prescription lies in its modification of the equilibrium potential $U_{\mathrm{eq}}\left(x\right)$, as described by Equation (8). In Figure 1a and Figure 2a, we illustrate how heterogeneous diffusion alters the potential. It is evident that for the same noise level, the parameter α changes the height of the potential barrier. Specifically, in the Hänggi–Klimontovich prescription (α=1), the barrier is the highest. Thus, the particle remains more time in the well, resulting in the smallest decay rate. Conversely, in the Itô prescription, the barrier is the lowest, leading to the greater decay rate since the particle can escape from the well more easily. This effect can be so pronounced that it modifies the entire structure of the potential for sufficiently large noise levels, as shown in Figure 1b and Figure 2b.

Equation (35) for the decay rate underscores the critical role of the equilibrium potential $U_{\mathrm{eq}}\left(x\right)$, which completely codifies the dependence of the dynamics on the stochastic discretization prescriptions. Our findings reveal that the choice of stochastic prescriptions directly influences decay rates, reflecting the underlying physics encoded in the critical points of the equilibrium potential and its local curvatures. Furthermore, the generalization to asymmetric potentials enriches the applicability of Kramers’s framework, accommodating a broader class of real-world systems.

Future investigations could extend this work by exploring higher-order corrections to the weak noise approximation and considering multidimensional landscapes. Such endeavors would further deepen our understanding of noise-induced transitions in complex systems.

## Data Availability

Data supporting reported results are available from the corresponding authors, D.G.B. and Z.G.A., upon reasonable request.
